# Employment and further study outcomes for care-experienced graduates in the UK

**DOI:** 10.1007/s10734-020-00660-w

**Published:** 2020-12-20

**Authors:** Neil Harrison, Zoë Baker, Jacqueline Stevenson

**Affiliations:** 1 Rees Centre, Department of Education, University of Oxford, Oxford, UK; 2 Centre for Development and Research in Education, Sheffield Hallam University, Sheffield, UK; 3 Lifelong Learning Centre, University of Leeds, Leeds, UK

**Keywords:** Care-experienced students, Care leavers, Graduate outcomes, Inequality, Widening access, Widening participation

## Abstract

Life outcomes for people who spent time in the care of the state as children (‘care-experienced’) are known to be significantly lower, on average, than for the general population. The reasons for this are complex and multidimensional, relating to social upheaval, disrupted schooling, mental and physical health issues and societal stigmatisation. Previous studies across several countries have demonstrated that they are significantly less likely to participate in higher education and more likely to withdraw early. However, little is currently known about their outcomes after graduation.

This paper therefore explores the initial outcomes for the 1,010 full-time students identified as care-experienced within the cohort graduating from an undergraduate degree programme in the UK in 2016/17—the most recent year for which data are available. They were found to be slightly more likely to be unemployed and less likely to be in work (and particularly professional work) than their peers, but, conversely, more likely to be studying. These differences largely disappeared once background educational and demographic factors were controlled.

The paper discusses the relationship between care-experience and other sites of inequality, concluding that care-experienced graduates are crucially over-represented in groups that are disadvantaged in the graduate labour market—e.g. by ethnicity, disability or educational history. This intersectional inequality largely explains their lower graduate outcomes. While there are important limitations with the data available, this speaks for the transformational potential of higher education in enabling care-experienced graduates to transcend childhood adversity. Recommendations for national policy and local practices conclude the paper.

## Introduction

In the United Kingdom (UK)^1^, the terms ‘looked after children’ or ‘children in care’ refer to those who are removed by local authorities with a ‘care order’ and placed in foster care, residential homes, or with extended family members; this is most commonly due to abuse or neglect within the birth family (Department for Education [DfE] 2019a). About 1% of the UK child population is in care at any one time. Adults who were in care at some point in their childhood are often referred to as ‘care-experienced’ and are the focus of this paper.

The legacy of childhood trauma can unsurprisingly impact educational outcomes for children in care and care-experienced adults. School attainment amongst care-experienced individuals is generally lower than the wider population (Sebba et al. 2015). Understandably, lower attainment can subsequently impact on higher education (HE) participation rates for those who are care-experienced and largely explains why they are severely underrepresented at this level of study. In England, the HE participation rate for care-experienced students is around 12% up to the age of 23, compared with 43% in the general population (Harrison 2017). Care-experienced students include both those transitioning into HE directly from care (often referred to as ‘care leavers’) and those returning in later adulthood; the latter group predominates, without around half being over 21 on entry (Harrison 2020).

The situation for care-experienced adults in the UK is echoed in many other countries, with a growing concern about their ability to access and thrive within HE, including in Australia (e.g. Harvey et al. 2015; Wilson, Harvey and Mendes 2019), the USA (e.g. Hernandez and Naccarato 2010; Okpych and Courtney 2019), Israel (e.g. Rafaeli and Strahl 2014), Ireland (e.g. Brady et al. 2019) and other European nations (e.g. Jackson and Cameron 2014).

While emerging literature has provided understandings of challenges and enablements in accessing and progressing through HE, there is a knowledge gap concerning graduate outcomes for care-experienced students. Other groups who are underrepresented in HE have been noted to encounter specific challenges when accessing the labour market and further study opportunities upon graduation (Behle 2016; Britton et al. 2016; Zwysen and Longhi 2018). Yet, the legacy of care could result in potentially unique disadvantages when transitioning out of HE. These may include an absence of informal support networks (or ‘social capital’) that can enhance access to employment opportunities (Stein 2012), unstable accommodation (Bengtsson et al. 2018; Centre for Social Justice [CSJ] 2019; Wilson et al. 2019), financial difficulties (Cotton et al. 2014; Gypen et al. 2017), mental/physical health issues (McNamara et al. 2019; O’Neill et al. 2019; Wilson et al. 2019) and societal stigma (Stein 2012). Despite most higher education institutions (HEIs) in the UK offering support for care-experienced students entering, and progressing through, their undergraduate studies, this often ends at or before graduation. There is little or no targeted support for care-experienced students’ graduate transitions, which can lead to high levels of anxiety over the future (Ellis and Johnston 2019). Such anxieties may affect how students make their plans for graduation, resulting in some options being closed off (Stevenson et al. 2020). To understand how care-experienced students who successfully enter and progress through HE fare after graduation, this paper uses the most recent national survey data available (from 2016/17) to provide the first insight into graduate outcomes for these students.

## Literature review

The individual benefits of HE have been well-documented by government and commentators. Such benefits include increased earning potential, lower likelihood of unemployment, better physical and mental health and avoidance of risk behaviours (Department for Business, Innovation and Skills, 2013; Johnstone 2014). This is important to highlight in the context of life outcomes for care-experienced individuals, as these have been widely reported as characterised by disadvantage. They are more likely to have lower earnings (Gypen et al. 2017), face higher rates of unemployment (Okpych and Courtney 2014), encounter difficulties with housing and homelessness (Häggman-Laitila et al. 2018), experience physical and mental health issues (Brännström et al. 2017; McNamara et al. 2019) and face stigma (Stein 2012). These disadvantages can be longstanding, with longitudinal research from Sweden finding that they persisted until middle age for over half of care-experienced adults (Brännström et al. 2017). The benefits of HE could therefore play a transformative role in life outcomes.

Even though HE has the potential to be transformative for care-experienced individuals, they are severely underrepresented (Harrison 2017). A number of reasons for this have been suggested. The proximal cause is undoubtedly that they are less likely to hold qualifications offering entry to HE (Jackson et al. 2005; Sebba et al. 2015)^2^. However, there are also important distal factors. For example, care-experienced people are over-represented among those with long-term health issues, such as disability or mental health difficulties (Department for Education and Skills [DfES] 2007; O’Neill et al. 2019), as well as in substance misuse and offending behaviour (Dixon 2007). These are often the result of circumstances that saw the child entering care, such as abuse and neglect (CSJ 2019; McNamara et al. 2019), or of experiences while in the care system itself. For instance, higher numbers of placement moves, which cause educational disruption, have been shown to have a detrimental impact on attainment (Jackson and Ajayi 2007; Sebba et al. 2015). This is compounded by low educational expectations from others (Jackson et al. 2005; Wilson et al. 2019) and non-prioritisation of education by social workers in organising placements (Jackson and Cameron 2012).

In the UK, care-experienced students in HE are more likely to be older, be women, identify as disabled, not be a UK national^3^, be from a minority ethnic group and attend lower status HEIs, having often progressed to HE via vocational routes and alternative pathways (Harrison 2020). Notably, care-experienced students are also 38% more likely to withdraw from their HE studies, all else being equal (Harrison 2017). The most commonly cited reason for withdrawal amongst care-experienced students is academic challenges (Cotton et al. 2014; Harrison 2017). This may partially be explained by the higher likelihood of care-experienced students entering HE via vocational pathways (Jackson and Ajayi 2007), where students are shifting from programmes that use more practical and competency-based learning approaches to ones in HE that valorise critical knowledge (Catterall et al. 2013). Other commonly reported reasons for withdrawal from HE for care-experienced students include mental health difficulties—particularly those that are complex and longstanding (Harrison 2017; Stevenson et al. 2020)—and financial issues (Cotton et al. 2014). In the face of such hardships, research has highlighted that support from a trusted individual and access to financial support is key to assisting in the transition into, and through, HE (Driscoll 2013). This is also required beyond HE, with Courtney (2019) noting that a continuation in support from social workers for longer could allow the facilitation of networking opportunities for care-experienced graduates, enhancing employment outcomes.

Until now, graduate outcomes for care-experienced students have been unresearched. Yet, the general literature on graduate outcomes in the UK highlights inequalities experienced by other underrepresented groups in HE; this provides some indications of what care-experienced graduates may encounter when transitioning to employment and/or further study. Those from socioeconomically disadvantaged backgrounds earn, on average, 10% less upon graduation than their more-advantaged counterparts (Britton et al. 2016). They are also less likely to be in professional employment following graduation (Higher Education Funding Council for England 2015). Students from minority ethnic groups are less likely to be employed 6 months after graduation, though differences in earnings are small (Zwysen and Longhi 2018). A number of factors contribute to these outcomes, including the following: discipline studied, with STEM graduates being more likely to find graduate work (Behle 2016), and be in receipt of higher earnings (DfE 2019b); prior attainment, with Britton et al. (2016) finding that much of the earnings difference amongst graduates is attributable to this; type of HEI attended, with those graduating from higher status^4^ HEIs being more likely to successfully access top professions (Ashley et al. 2015); and degree classification, with those awarded first and upper-second class degrees being more likely to enter ‘high skilled’ employment (DfE 2019b). There are similar inequalities evident in progression to postgraduate study, with students from lower socioeconomic groups, those that studied at post-1992 institutions, and those studying ‘applied’ subjects (such as Education) as opposed to ‘pure’ subjects (e.g. Science) being less likely to progress to higher degrees (Wakeling and Hampden-Thompson 2013).

These inequalities provide some speculative insights into what constraints care-experienced graduates may face. For example, lower levels of prior attainment amongst care-experienced students (Sebba et al. 2015) may mean that they are likely to earn less following graduation (Britton et al. 2016). Furthermore, care-experienced students are more likely to study social sciences and creative arts subjects , less likely to study STEM subjects and are underrepresented in higher status universities (Harrison, 2020). This may mean that they are less likely to (a) locate graduate-level employment (Behle 2016), (b) progress to postgraduate study (Wakeling and Hampden-Thompson 2013) and (c) access high-skilled employment (DfE 2019b).

## Methodology

All students graduating from UK universities are surveyed around 6 months after the end of their course to identify what activities they are currently engaged in—work, further study, unemployment or other activities. Known as the Destinations of Leavers from Higher Education (DLHE) dataset, it is made available to researchers on an anonymised basis. This study uses the 2016/17 DLHE data; the most recent available and the last to be collected under this methodology^5^. Over three-quarters of graduates complete the survey, and the data are generally considered to be robust and representative of the graduate population (Higher Education Statistics Agency [HESA], 2019).

Furthermore, the DLHE data can be linked at the individual level to other data held about graduates by HESA, providing a rich opportunity for multivariate analysis. Table 1 provides a full list of linked variables and the categorisations used in this study.

**Table 1:** List of variables used in this study

Variable	Definition	Notes
Sex	Male/female	Predates the use of ‘non-binary’ category
Age on entry	20 or under/21 to 24/25 to 29/30 and over	
Disability	Not disabled/Disabled and receiving the Disabled Students’ Allowance/Disabled, but not receiving the Disabled Students’ Allowance	Disability data is collected from students on the basis of self-declaration following the definition in the Equality Act 2010 and therefore covering, *inter alia*, physical impairments, long-term health conditions, mental health issues and specific learning difficulties. The Disabled Students’ Allowance (DSA) covers study-specific (i.e. not living) costs associated with a disability. Those not receiving the DSA tend to include those with mental health issues and chronic illness, although assessments are undertaken individually
Ethnicity	White/Black (inc. African and Caribbean)/Indian, Pakistani or Bangladeshi/Other Asian (inc. Chinese)/Mixed Heritage/Other or not known	Because of low numbers, it was necessary to combine ethnic groups. Various configurations were explored, but they did not materially impact on the analysis
Nationality	UK/Other nationality	See Footnote 3
Care status	Care-experienced/Not care-experienced	
Entry qualifications	Previous degree/Previous undergraduate study (e.g. certificate or diploma)/Level 3 (< 80 tariff points)/Level 3 (80 to 159 tariff points)/Level 3 (> 159 tariff points)/Other Level 3/Other (Level 2, no qualifications or unknown)	Level 3 qualifications offer entry to HE and are allotted ‘tariff points’ based on their level and intensity. It is possible with work experience to gain admission without Level 3 qualifications
HEI type	Russell Group/Other pre-1992 provider/Other provider	See Footnote 4
Subject of study	Natural sciences/Healthcare/Mathematics, engineering and construction/Computer science and technology/Social sciences/Law, business and communications/Languages, history and philosophy/Creative arts/Education	Derived from the ‘JACS’ codes used to categorise courses in the UK
Degree class	First/Upper Second/Lower Second/Third or Pass/Unclassified	‘Unclassified’ relates to courses that do not award classifications—e.g. medicine
Sandwich year	Yes/No	An academic year spent studying in another country or working in industry
Principal activity	Full-time work/Part-time work/Work mainly, plus study/Study mainly, plus work/Full-time study/Part-time study/Due to start work shortly/Unemployed/Other	Derived from the DLHE survey. ‘Other’ includes travelling, caring responsibilities, long-term sickness and similar
Status of work	Managers, directors and proprietors/Professionals/Associate professionals/Administrative and secretarial/Skilled trades, personal service and leisure/Customer service and sales occupations/Machine operatives and elementary occupations	Derived from the DLHE survey
Level of study	Research postgraduate/Taught postgraduate masters/Postgraduate certificate or diploma/Undergraduate/Professional qualification/Other or informal qualification	Derived from the DLHE survey

Importantly, this includes a marker for care-experienced status, derived in part from self-declarations made on application or entry to HE and in part from information informally collected by HEIs about students when they are on course—e.g. when a student makes an application for a bursary. This marker undoubtedly contains some false positives (e.g. people mistakenly considering themselves to be care-experienced), false negatives (e.g. people choosing not to disclose their care status) and other forms of bias (e.g. differential recording between HEIs), but it is nevertheless the most reliable data currently available on graduates’ care status; see Harrison (2020) for a fuller discussion.

Several exclusions were made from the dataset before analysis. Firstly, international graduates were excluded as very little information is available on care-experience in other countries. Secondly, only previous full-time undergraduates were included, as data on care status for part-time and postgraduate students is very partial. Thirdly, graduates of sub-degree courses were excluded as they tended to have very different experiences, with most progressing into degree programmes. Fourthly, graduates for whom the care status marker was missing entirely were excluded as it was not known whether they were care-experienced or not; this is a heterogeneous group of students entering outside of the normal HE application process and including, *inter alia*, some students on work-based learning courses and direct admission schemes (Harrison 2020). These exclusions are discussed in more detail in the ‘Limitations’ section below.

The final inclusion category was that the graduate had completed the DLHE survey. The response rate in the amended dataset for care-experienced graduates (72%) was slightly lower than for other graduates (76%). The final dataset used for analysis comprised 171,680 graduates, of whom 1,010 (0.6%) were identified as care-experienced^6^.

The study takes a three-stage approach to analysis. The first stage provides a bivariate descriptive analysis of care-experienced graduates compared with other graduates. The second examines headline outcomes (work, further study and unemployment) for the two groups. The third uses logistic regression analysis to explore multivariate differences in outcomes between care-experienced and other graduates. Logistic regression allows for outcomes to be compared while holding a range of potential predictor variables constant and thereby seeking to isolate the specific contribution of care-experience to overall outcomes, all else being equal (Field 2017). SPSS v25 was used for analysis, and a 5% significance level was used.

This paper has its origins in an earlier research project (Stevenson et al. 2020). The first two analytical stages outlined above are summarised from that project to provide essential context, but the third is unique to this paper and forms the basis of the subsequent discussion.

## Findings

### Graduate cohorts

Table 2 shows the demographic profile of the care-experienced graduate sample in comparison with other graduates. Several key differences are readily apparent. Care-experienced graduates had a higher preponderance to be disabled (24.7%, compared with 14.2%) and to be a non-UK national (16.3%, compared with 8.2%). They were older, on average, being over twice as likely to be aged 25 or over on entry (22.7%, compared with 9.2%). They were also disproportionately drawn from the Black, Other Asian and Mixed Heritage ethnic groups.

**Table 2:** Demographic profile, by care status

		Care-experienced	Not care-experienced
Sex	Female	60.4%	57.2%
	Male	39.6%	42.8%
Age on entry	20 and under	27.5%	41.5%
	21 to 24	49.9%	49.3%
	25 to 29	9.5%	4.1%
	30 and over	13.2%	5.1%
Disability	Disabled and receiving DSA	15.1%	7.8%
	Disabled, but not receiving DSA	9.6%	6.4%
	Not known to be disabled	75.3%	85.8%
Ethnicity	White	63.2%	78.1%
	Black (inc. African and Caribbean)	14.9%	5.5%
	Indian, Pakistani or Bangladeshi	5.7%	8.3%
	Other Asian (inc. Chinese)	5.4%	2.6%
	Mixed heritage	7.5%	3.7%
	Other/not known	3.2%	1.8%
Nationality	UK national	83.7%	91.8%
	Other nationality	16.3%	8.2%

Table 3 presents a similar analysis for education variables. Care-experienced graduates entered HE with weaker entry qualifications overall, also notably making more extensive use of alternative qualifications (e.g. Level 3 without tariff points) or prior experience. They were substantially less likely to attend Russell Group and other pre-1992 universities, which was partly—but not entirely—due to having lower entry qualifications (see Stevenson et al. 2020 for details on this point). There were modest differences in subjects studied between care-experienced and other graduates, with the former being somewhat over-represented in social sciences and creative arts, but underrepresented in natural sciences, technology-based subjects and the humanities, and among those taking a sandwich year. Importantly, care-experienced graduates were somewhat less likely to have received first or upper-second class degrees, although a clear majority still did so (70.1%, compared with 79.8%); this could almost entirely be attributed to differences in entry qualifications and demographic profile.

**Table 3:** Educational profile, by care status

		Care-experienced	Not care-experienced
Entry qualifications	Previous UG study	11.2%	8.7%
	Level 3—fewer than 80 tariff points	11.5%	5.8%
	Level 3—80 to 159 tariff points	48.1%	53.3%
	Level 3—160 or more tariff points	15.6%	27.3%
	Level 3—other	10.3%	3.6%
	Other (level 2, no qualifications or unknown)	3.4%	1.3%
Institutional type	Russell Group university	10.5%	24.4%
	Other pre-1992 university	15.7%	18.7%
	Other provider	73.8%	56.9%
Area of study	Natural sciences	15.9%	20.0%
	Healthcare	5.9%	5.6%
	Maths, engineering and construction	5.4%	7.7%
	Computer science and technology	5.2%	5.0%
	Social sciences	18.9%	11.4%
	Law, business and communications	21.9%	20.0%
	Languages, history and philosophy	6.6%	11.4%
	Creative arts	14.9%	12.8%
	Education and combined studies	5.2%	6.0%
Degree class	First class	20.3%	27.8%
	Upper second class	49.8%	52.0%
	Lower second class	23.2%	16.1%
	Third class or Pass	5.3%	2.2%
	Unclassified or not applicable	1.4%	1.9%
Sandwich year	Yes	5.4%	8.0%
	No	94.6%	92.0%

This first stage of analysis thus confirmed care-experienced graduates as having a distinct profile compared with the general population. They are more likely to be older, disabled, from a minority ethnic community and a non-UK national—all potential markers for educational disadvantage. In addition, they tended to have entered HE with lower entry qualifications, attended a lower status HEI and been awarded a lower classification of degree, although the former trends largely explained the latter. These patterns are consistent with previous studies of data on care-experienced students (e.g. Harrison 2017, 2020) and more broadly with the care population (DfE 2019a).

### Graduate outcomes

**Fig. 1:**
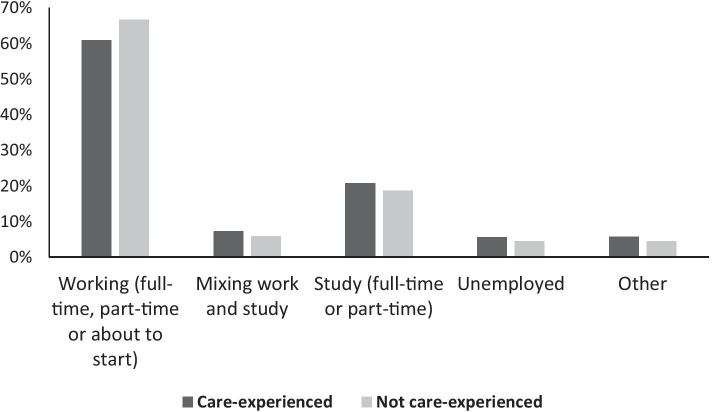
Main activity 6 months after graduation, by care status

In the DLHE survey, graduates are asked to provide information about their current activities 6 months after graduation. These are coded into consistent categories before being made available to researchers (HESA 2019). Due to small numbers in some categories, the nine ‘main activity’ categories listed in Table 1 have been collapsed into five: working, studying, mixing work and study, unemployed and other. The activities for care-experienced and other graduates are illustrated in Fig. 1.

The overall patterns of activity are similar between the groups. Graduates who were not care-experienced were somewhat more likely to be in work alone (66.6%, compared with 60.9%), while care-experienced graduates were disproportionately found in the other four categories. Including those mixing activities, 27.9% of care-experienced graduates had moved on to further study; the equivalent figure for other graduates was 24.6%. Care-experienced graduates were also slightly more likely to be unemployed (5.5%, compared with 4.4%) or engaged in ‘other’ activities.

**Fig. 2:**
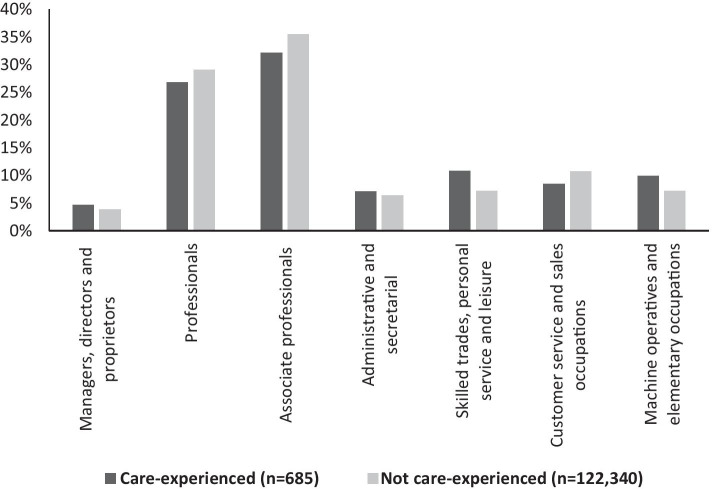
Occupational classification of graduates in work, by care status

Turning to look in more depth at those who are working, Fig. 2 shows the breakdown into the Standard Occupational Classification used in the UK to hierarchically categorise work by status; the standard nine-point system has been collapsed due to small numbers in two categories. Once again, the profile between care-experienced and other graduates is similar. A commonly used distinction between ‘professional’ (the first three classes) and ‘non-professional’ (the remainder) work shows 63.7% of care-experienced graduates in professional roles, compared with 68.5% of other graduates; restricting to those in full-time work only, the figures are 70.7% and 77.0% respectively. In other words, care-experienced graduates were somewhat less likely than their peers to be in professional roles 6 months after graduation, although the majority were. Care-experienced graduates were disproportionately likely to be working in public administration, social work and residential care, but less likely to be in the financial, legal or accounting industries; the overall salary profiles between care-experienced and other graduates were almost identical (see Stevenson et al. 2020 for details).

**Fig. 3:**
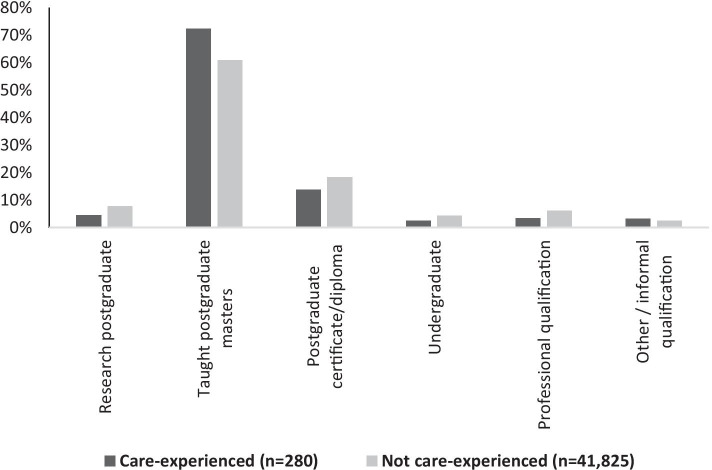
Further study activity 6 months after graduate, by care status

Figure 3 shows the level of further study for those studying 6 months after graduation. Care-experienced graduates were substantially more likely to be undertaking a taught master’s degree than other graduates (72.3%, compared with 60.8%), but less likely to be pursuing a research degree^7^, postgraduate certificate/diploma or professional qualification.

Finally, we introduce a compound measure for a ‘positive graduate outcome’ derived from the complex data outlined above. We take a positive graduate outcome to be *either* (a) working in a professional role *and/or* (b) studying on a postgraduate or professional course. In constructing this measure, we exclude graduates who report ‘other’ activities as they are effectively absent from the labour market for heterogeneous reasons (e.g. travelling). We appreciate that this is a considerable simplification. For example, some people in professional roles may not be in their preferred career, with others undertaking informal study for well-founded reasons. Also, the timescale of 6 months is one in which many graduates are ‘finding their feet’ and taking time to review their options. Nevertheless, for the purposes of comparing overall immediate outcomes for care-experienced and other graduates, we argue that the measure has utility. Using this compound measure, we find that 70.1% of care-experienced graduates had a positive graduate outcome, compared with 72.3% of other graduates; this difference was not statistically significant. By definition, the remainder were unemployed, in non-professional work or studying an additional undergraduate or informal course.

To summarise, care-experienced graduates had broadly similar outcomes to other graduates—notably, this is despite having attended lower status HEIs and achieving lower degree results, on average. They were somewhat less likely to be working and to be in professional work, but conversely more likely to be studying. Their unemployment rate was slightly higher, although it was generally low for both cohorts.

### Predictors for graduate outcomes

The third stage of analysis continues to use the positive graduate outcome measure defined in the previous section. It becomes the dichotomous dependent variable in a series of three binary logistic regression models in Table 4. Model 1 includes only the care status marker. Model 2 adds the educational variables defined in Table 1, and Model 3 further adds the demographic variables.

**Table 4:** Binary logistic regression models for positive graduate outcomes

	Model 1		Model 2		Model 3	
	B (SE)	OR (*p*)	B (SE)	OR (*p*)	B (SE)	OR (*p*)
Care-experienced (ref = no)						
Yes	− .106 (.071)	.899 (.135)	.071 (.073)	1.073 (.330)	.057 (.073)	1.059 (.431)
Degree class (ref = first class)						
Upper second class			− .535 (.014)	.586 (< .001)	− .519 (.014)	.595 (< .001)
Lower second class			− .992 (.018)	.371 (< .001)	− .981 (.018)	.375 (< .001)
Third class or pass			− 1.508 (.036)	.221 (< .001)	− 1.511 (.037)	.221 (< .001)
Unclassified			.009 (.050)	1.009 (.863)	− .076 (.051)	.926 (.132)
HEI type (ref = other)						
Russell Group university			.380 (.015)	1.462 (< .001)	.402 (.015)	1.494 (< .001)
Other pre-1992 university			.144 (.016)	1.154 (< .001)	.169 (.016)	1.185 (< .001)
Subject (ref = education/combined)						
Natural sciences			− .471 (.027)	.624 (< .001)	− .474 (.028)	.622 (< .001)
Healthcare			.181 (.037)	1.199 (< .001)	.184 (.038)	1.202 (< .001)
Maths, engineering and construction			− .077 (.033)	.926 (.021)	− .105 (.034)	.900 (.002)
Computer science and technology			− .206 (.036)	.814 (< .001)	− .244 (.037)	.784 (< .001)
Social sciences			− .455 (.029)	.635 (< .001)	− .466 (.030)	.627 (< .001)
Law, business and communications			− .427 (.027)	.653 (< .001)	− .417 (.028)	.659 (< .001)
Languages, history and philosophy			− .600 (.030)	.549 (< .001)	− .601 (.030)	.549 (< .001)
Creative arts			− .634 (.028)	.531 (< .001)	− .635 (.028)	.530 (< .001)
Sandwich year (ref = no)						
Yes			.502 (.025)	1.652 (< .001)	.480 (.025)	1.616 (< .001)
Sex (ref = female)						
Male					.111 (.012)	1.117 (< .001)
Ethnicity (ref = white)						
Black (inc. African and Caribbean)					− .053 (.025)	.948 (.032)
Indian, Pakistani or Bangladeshi					− .103 (.021)	.902 (< .001)
Other Asian (inc. Chinese)					− .187 (.035)	.829 (< .001)
Mixed heritage					− .093 (.029)	.911 (.002)
Other or not known					− .094 (.042)	.910 (.026)
Age on entry (ref = 20 or under)						
21 to 24					.077 (.012)	1.080 (< .001)
25 to 29					.298 (.031)	1.348 (< .001)
30 or over					.392 (.029)	1.480 (< .001)
Disabled (ref = not disabled)						
Disabled and receiving DSA					− .002 (.021)	.998 (.936)
Disabled, but not receiving DSA					− .057 (.023)	.944 (.012)
Nationality (ref = UK)						
Not UK					− .166 (.021)	.847 (< .001)
Constant	.852 (.071)	2.344 (< .001)	1.709 (.027)	5.522 (< .001)	1.620 (.028)	5.054 (< .001)
Nagelkerke *R*^2^	< .001		.066		.070	

Model 1 shows care-experienced graduates as being somewhat less likely than otherwise similar graduates to have a positive graduate outcome after 6 months, with an odds ratio of less than one, but this is not statistically significant (*p* = .135). This is consistent with the analysis in the previous section.

However, and interestingly, once the educational variables are entered in Model 2, the odds ratio for care-experienced graduates rises above 1. This indicates that once degree subject, HEI, degree classification and sandwich years are taken into account, care-experienced graduates are actually slightly more likely than other graduates to report a positive outcome, although this is again not statistically significant (*p* = .330). Model 3, which additionally controls for sex, ethnicity, nationality, disability status and age, provides a very similar picture, with care-experienced graduates having slightly better outcomes, on average, but at a level that is not statistically significant (*p* = .431).

We can conclude from this analysis that being care-experienced is not in itself a predictor for less positive graduate outcomes; if anything, Models 2 and 3 suggest that the opposite is true, albeit marginally. Rather, care-experienced graduates are notably over-represented in various educational and demographic groups that *are* markedly less likely to have a positive graduate outcome—i.e. achieving a lower degree classification, attending a lower status HEI, being from a minority ethnic community, being a non-UK national and being disabled. In other words, care-experienced graduates do as well—on average and within the timeframe dictated by the dataset—as other students with a similar profile.

Finally, we note that the *R*^2^ statistics, indicating the proportion of the variance explained by the models and therefore their ability to predict an individual’s outcomes based on the variables listed, are relatively low. This can have two meanings. Firstly, there may be important unobserved variables missing from the model which have greater explanatory power than those included. With respect to graduate outcomes, this might include personal characteristics for which no data are available, such as motivation, job-searching skills or desire to wait for the right job/study opportunity. Secondly, there may be a high degree of underlying randomness concerning which graduates are able to secure positive outcomes in the timeframe, based on the vagaries of the labour market. It is impossible to resolve these possibilities with the data available. However, the key point is that there is no evidence herein to conclude that care-experienced graduates are systematically disadvantaged beyond their over-representation in groups which are known to be disadvantaged.

## Discussion

As outlined earlier, care-experienced students are likely to face a number of challenges when progressing into and through HE, including (but not limited to) low levels of prior attainment (Sebba et al. 2015), disability and physical/mental health issues (O’Neill et al. 2019), financial difficulties (Cotton et al. 2014) and engagement in risk behaviours (Dixon 2007). Despite this, we have found that care-experienced graduates do not have significantly lower outcomes than other graduates with the same educational and demographic profile—indeed, they are slightly higher.

This is something of an unexpected finding. As noted above, the literature has presented a number of structural challenges faced by care-experienced adults in both the education and employment spheres. Some of these are potentially unique to care-experienced people. For example, Stein (2012) explores the societal stigma, stereotyping and discrimination resulting from being care-experienced (also see Wilson et al. 2019), while Dickens et al. (2014) examine the implications for transitions in the absence of family ‘safety nets’ for housing and finance. With particular reference to HE, Ellis and Johnston (2019) report that the discontinuation in support provided by HEIs when care-experienced students are approaching graduation is a source of additional anxiety.

The majority of studies, however, find intersectional effects between care-experience and other sites of inequality that are somewhat better understood. For instance, in Barn et al. (2005) study of care leavers’ transitions to adulthood, young people from minority ethnic groups faced racial discrimination in the workplace, which then had negative repercussions for future employment. This is also likely to impact on care-experienced non-UK nationals (including asylum seekers and refugees), in addition to language barriers, unfamiliarity with UK work culture and application processes (Stevenson and Willott 2007; Robinson and Williams 2017). Similarly, being disabled can impact on fitness to find and engage in employment in itself, and disabled people can face discrimination by potential employers, resulting in HE to labour market transitions being ‘disabling experiences in themselves’ (Piggott and Houghton 2007, 585). In O’Neill et al. (2019) survey of care-experienced students, those identifying as disabled reported more difficulties with accommodation, finances and trauma recovery.

This is consistent with our findings. Care-experienced graduates are notably overrepresented in groups that *do* have markedly lower graduate outcomes, such as some disabled people, minority ethnic communities and non-UK nationals. In other words, the negative pressures on care-experienced students work primarily through these other sites of inequality, which will be shared to a greater or lesser extent with other students who are not care-experienced. For example, racial discrimination in the recruitment process or the lack of geographical mobility arising from disability are likely to impact on many graduates—rather, it is that care-experienced students have a constellation of overlapping and additive forms of disadvantage derived from their intersectional membership of multiple groups.

This is not to argue that there are *not* distinct, or even unique, forms of disadvantage associated with being care-experienced. However, within the data available to this study, their impact is muted, with even the suggestion that once the other forms of disadvantage are controlled for, care-experienced graduates are doing slightly better than average. One possible explanation is that, while many do experience stigma or other disadvantages, some care-experienced graduates are able to draw on additional personal resources that actively assist them in moving into work or further study. These may be unrecognised by the individual, but discourses around heightened resolve, motivation and determination to succeed appear frequently in the literature (Harrison 2017; O’Neill et al. 2019). Another explanation is that care-experienced students who complete their HE studies may have had positive experiences whilst in care that result in them successfully ‘moving on’, for instance, experiences of stability, continuity, positive relationships and educational success (Stein 2012). These attributes and experiences could ameliorate the other care-related barriers to successful graduate transitions, although it should be remembered that the unemployment rate *is* somewhat higher for care-experienced graduates. In addition, care-experienced students tend to be markedly older than average when entering HE and our analysis suggests that increasing age is a predictor for positive graduate outcomes, possibly due to more prior experience of engaging with the labour market, greater accumulated social capital and a clearer career vision. This particular intersectionality therefore tends to act as a protective factor for care-experienced graduates.

While care-experienced graduates are not less likely to have positive outcomes overall, the proportion in *professional* employment was considerably lower. However, there are particular challenges associated with being care-experienced that can impinge on graduate transitions, leading to these being ‘compressed’ (Stein 2012). Lacking family safety nets, care-experienced graduates may be more preoccupied with fulfilling their basic needs as quickly as possible through prioritising obtaining accommodation and locating a source of income (Dickens et al. 2014) after graduation. Instability in the labour market coupled with affordable housing shortages mean that there is increased reliance on family support for young people generally (Gypen et al. 2017). Opportunities for ‘yo-yo transitions’ (Bengtsson et al. 2018)—where graduates return to live in the parental home—are unavailable for care-experienced graduates, resulting in a lack of time and psychological space to formulate future plans (Stein 2012). An absence of family support also restricts the size of care-experienced graduates’ social networks; this, in turn, limits access to useful information about employment opportunities (Clarke 2018), placing them at a further disadvantage. This could lead to more dynamic and temporary outcomes (Brännström et al. 2017), with some care-experienced graduates feeling pressure to locate any form of employment immediately rather than professional employment specifically. Of course, this may not be the case for all care-experienced graduates, with those who continue to receive contact and support from former carers potentially experiencing more stable transitions out of HE (Stein 2012); Optych and Courtney (2019) argue that extended support periods are helpful to underpin educational outcomes.

This absence of safety nets for care-experienced graduates could also help to explain why they are more likely to progress to postgraduate study. Undergraduate study can initially provide a period of welcomed stability, with many HEIs offering generous support packages including bursaries and year-round accommodation (Ellis and Johnston 2019; Stevenson et al. 2020), as well as an opportunity for a fresh start and the chance to build a new identity. Ellis and Johnston (2019, 7) found that while many care-experienced students had faced profound challenges in HE, they often nevertheless felt ‘that they found a place that they belonged’. Although financial and accommodation support from HEIs typically ends on completion of their undergraduate studies, progressing to postgraduate programmes could extend some sense of stability for care-experienced graduates, with emotional and other forms of support via professional staff and friendship networks within the HEI. Indeed, some students in Ellis and Johnston’s (2019) study talked about their anxieties about moving on. Postgraduate study can therefore enable a more gradual transition from the supportive undergraduate environment to a more independent graduate life.

Apprehensions over future insecurity could also increase the importance of HE ‘paying off’ for care-experienced graduates. As for other underrepresented groups, HE might provide ‘reassurance’ by increasing the chances of achieving stability by reducing the risk of long-term unemployment (Harrison 2019). Yet, for care-experienced students, the absence of safety nets means their need for ‘pay off’ is arguably higher. Engaging in postgraduate study may therefore be viewed as a way of increasing their chances in the graduate employment market; we explore this further in Baker, Harrison, Wakeling and Stevenson (forthcoming). In addition, care-experienced students often express how their own childhood experiences motivate them to work in caring professions such as social work (Stevenson et al. 2020); these careers often necessitate a postgraduate qualification for entry. There is potentially, therefore, a double ‘push’ towards postgraduate study—remaining within a supportive and predictable environment while mitigating fears about securing work; the latter may be exacerbated by continuing mental health issues.

Finally, the propensity for care-experienced individuals to enter HE via alternative routes, such as vocational courses (Harrison 2017, 2020; Jackson et al. 2005), appears to result in some enduring effects on their graduate outcomes. Those entering HE with vocational qualifications are more likely to attend lower-status HEIs (Masardo and Shields 2015). They are also less likely than those with traditional entry qualifications to be awarded a first or upper-second class degree, particularly in the Russell Group (Shields and Masardo 2017); these factors reduce graduates’ chances of entering professional employment (Ashley et al. 2015; DfE 2019b).

Why, then, do many care-experienced students opt to enter HE via alternative routes? Firstly, those undertaking vocational qualifications are more likely to be from minority ethnic groups and to be disabled (Kelly 2017). As care-experienced individuals are over-represented in these groups, this can provide one explanation. Importantly though, some care-experienced people pursue vocational qualifications in an attempt to enter employment more quickly to become self-supporting as soon as possible upon leaving care (Jackson and Cameron 2012). Others report that they were encouraged to take these pathways by carers, which is indicative of others’ low educational expectations (Jackson et al. 2005). Perhaps one of the most likely reasons for this though is that low levels of attainment in compulsory schooling arising from instability, placement moves and the legacy of trauma (Jackson and Ajayi 2007; Jackson et al. 2005; Sebba et al. 2015) lead to reduced options in post-compulsory education. This can limit HE pathways later, with a lack of parity across qualifications afforded by more selective HEIs which can translate to exclusionary admission processes (see Baker 2019). Therefore, it is not necessarily that care-experienced students actively choose such routes into HE; rather, their options are constrained by circumstances arising from their care histories. This subsequently largely explains the difference in headline figures for positive graduate outcomes—a culmination of accumulated disadvantage that can be traced back, directly or more indirectly, to the individual’s entry into care.

## Limitations

The principal limitation to this study is our use of a secondary national dataset over which we have no control. While generally robust, it has several weaknesses outlined in our methodological discussion. No reliable data yet exist for part-time, postgraduate or international students, and we have had to exclude those entering full-time degree programmes through various alternative routes where no data on their care status exist. This latter exclusion is particularly unfortunate as we would expect care-experienced people to be over-represented in this group. Similarly, no data exist for people completing their degree in further education colleges as they are not included in the DLHE survey. It is, of course, possible that care-experienced graduates in these groups are substantively different from those in our study.

We also need to be mindful that we are only presenting the outcomes for those who *did* complete. The chances of care-experienced students completing their studies is substantially lower than otherwise similar peers due to the additional challenges they face (Cotton et al. 2014; Ellis and Johnston 2019; Harrison 2017; O’Neill et al. 2019; Stevenson et al. 2020)—Optych and Courtney (2019) find similarly high attrition rates in the USA. This ‘survivor effect’ means that some care-experienced students facing the most profound challenges will have left HE early and therefore be absent from our dataset. This could make our findings appear more positive than reality.

Finally, and perhaps most significantly, we only have data on the first 6 months following graduation: a period traditionally associated with labour market turbulence. It is possible that real inequalities emerge for care-experienced graduates *after* this timeframe—for example, with enduring mental or physical health issues impacting on their opportunities for promotion. The recent shift to a 15-month survey period (see footnote 5) may provide different insights once these data become available.

## Conclusions

This first insight into the outcomes of care-experience graduates offers a promising picture. While it is based solely on a UK dataset, we believe that our findings will be useful to researchers and policymakers in other countries, given the ubiquity of concerns about how those taken into care as children fare in adulthood. Indeed, this form of intersectional analysis might usefully be extended to better understand outcomes for other disadvantaged social groups—e.g. student carers or student parents.

We have found that care-experienced students who successfully complete HE are not disadvantaged beyond their over-representation in other known disadvantaged groups. Although their unemployment rate is slightly higher than average, this is still very low. Additionally, their slightly lower likelihood to be in employment 6 months after graduation is balanced by their higher propensity towards further study. This points to the transformational nature of HE for care-experienced students, affording them scope to transcend the manifest disadvantages of their early lives.

While outcomes are promising for care-experienced graduates, this is not to say that accomplishing these is without its challenges. Care-experienced graduates are over-represented in known disadvantaged groups, and structural challenges faced by individuals within these groups who are not care-experienced will also be encountered by those who are. Importantly, reducing inequalities in access to HE for these groups will also significantly benefit care-experienced people. However, unique obstacles to achieving positive graduate outcomes that arise from being care-experienced persist, such as a lack of secure onward accommodation (CSJ 2019), financial difficulties (Cotton et al. 2014), stigma about care (Stein 2012), an absence of emotional support (Stevenson et al. 2020) and limited social networks (Stein 2012) which can restrict access to information about employment opportunities (Clarke 2018); these can all make graduate transitions a period that is fraught with anxiety and instability and may explain why care-experienced graduates are somewhat more likely to find themselves unemployed or in non-professional work. These concerns are likely to be heightened as a result of the global Covid-19 pandemic.

There is, therefore, a need to develop policy and practice targeted specifically at care-experienced graduates to assist with transitions out of HE. One perhaps easily implemented recommendation is for HEIs to promote the success of care-experienced graduates to students early on in their HE journeys; communicating this success may work to reduce HE withdrawal rates amongst care-experienced students and could be reassuring to those who have concerns when approaching completion of their studies. In order to create time and space for care-experienced graduates to transition into employment, HEIs could provide ‘exit’ bursaries and extend accommodation contracts for final year students past the point of completing their course. This would help to reduce immediate financial and housing pressures, affording the psychological space for care-experienced graduates to formulate future plans (Stein 2012). In turn, this would help to reduce the pressure to locate *any* form of employment immediately to fulfil their basic needs (Dickens et al. 2014), rather than professional employment, as these needs will be met in the immediate post-graduation phase. Additionally, focused alumni networks for care-experienced graduates should be developed to enable the continuous provision of careers-related information and guidance, increasing graduates’ knowledge of pathways into professional employment and helping to reduce unemployment rates. This will also help care-experienced graduates maintain a sense of connection to their HEI, reducing feelings of isolation as well as apprehension over the sudden loss of HE-provided support following graduation (Ellis and Johnston 2019; Stevenson et al. 2020).

Finally, given the importance of entry qualifications on subject choice, type of HEI attended, degree classification and, ultimately, graduate outcomes, more work needs to be done to ensure that care-experienced students are not defaulted into vocational and other alternative pathways at 16. The use of more inclusive and flexible admission policies, such as a willingness to accept (and crucially, support) those with vocational qualifications, will widen participation for many under-represented groups, *including* care-experienced students—particularly within higher-status HEIs. This study demonstrates that when given the opportunity, care-experienced students have every chance of thriving through HE and after graduation.

## Data Availability

The data used was purchased from the Higher Education Statistics Agency.

## Footnotes

1 The legislative framework, terminology and practices vary to some extent between the four constituent nations of the UK. For simplicity, the English variations are used herein.

2 In the UK, qualifications generally offering entry to HE are designed as Level 3. Most commonly, these are academically focused A Levels, but there is also a wide range of vocational qualifications available.

3 In this context, this includes students who hold a nationality other than British, but who have the right to study in the UK as a ‘home’ student rather than an ‘international’ student. This will include, but not be limited to, those with settled status, those from recent migrant families, asylum-seekers and refugees.

4 In 1992, HEIs that were formerly polytechnics were granted university status. Having university status prior to this date is therefore seen as a marker of higher status, although the distinction is increasingly eroded. The Russell Group is a self-identifying group of research-intensive universities that constitutes those with the highest status.

5 The new Graduate Outcomes survey uses a broadly similar approach, but leaves a fifteen-month period between graduation and data collection to allow more time for graduates to have found their way into career-oriented work.

6 Under HESA’s anonymity and disclosure requirements, all counts are rounded to the nearest five.

7 Direct progression from an undergraduate degree to a research degree is more common in the natural sciences, where care-experienced students are under-represented.
